# Economic evaluation of stent retrievers in basilar artery occlusion: An analysis from Chinese healthcare system perspective

**DOI:** 10.1371/journal.pone.0294929

**Published:** 2023-11-30

**Authors:** Hailong Xia, Qi Yang, Qibo Wang, Jielin Jia, Xipeng Liu, Shu Meng

**Affiliations:** 1 Department of Neurosurgery, Chongqing Red Cross Hospital(Jiangbei District People’s Hospital), Chongqing, China; 2 Department of Cardiology, The First Affiliated Hospital of Chongqing Medical University, Chongqing, China; 3 Department of Orthopaedic, Chongqing Red Cross Hospital(Jiangbei District People’s Hospital), Chongqing, China; 4 Internal medicine department, Chongqing Red Cross Hospital(Jiangbei District People’s Hospital), Chongqing, China; Foshan Sanshui District People’s Hospital, CHINA

## Abstract

**Purpose:**

This study aimed to investigate the cost-effectiveness of stent retriever (SR) versus best medical management (BMM) in patients with basilar artery occlusion (BAO) in China.

**Methods:**

We used a two-step approach to compare the cost-effectiveness of SR plus BMM with that of BMM alone over 20 years. A decision tree was initially constructed for the first 3 months, followed by a Markov model for the subsequent period. Collected data on clinical aspects were extracted from the BAOCHE investigation, while costs-related information was sourced from previously published research. The key metric for evaluating the primary outcome was the incremental cost-effectiveness ratio (ICER), achieved $/QALY. The threshold for identifying SR as highly cost-effective was set at an ICER below $12,551/QALY, SR was deemed cost-effective if the ICER ranged from $12,551 to $37,654 per QALY. Uncertainty was addressed using scenario, one-way sensitivity, and probabilistic sensitivity analyses (PSA).

**Findings:**

For Chinese patients with BAO, the 20-year cost per patient was $8678 with BMM alone and $21,988 for SR plus BMM. Effectiveness was 1.45 QALY for BMM alone, and 2.77 QALY for SR plus BMM. The ICER of SR + BMM versus BMM alone was $10,050 per QALY. The scenario and one-way sensitivity analyses revealed that in certain situations the ICER could exceed $12,551 per QALY, but remain below $37,654 per QALY. Results from the PSA suggested that SR was likely to be cost-effective for Chinese patients with BAO, with a probability exceeding 98% when considering a willingness-to-pay (WTP) threshold of $12,551 per QALY.

**Implications:**

Our study indicates that SR is an intervention option that is highly likely to be cost-effective for Chinese patients with BAO, with a probability of over 98% under the current WTP threshold of $12,551 per QALY.

## Introduction

Stroke is a leading cause of death and disability worldwide, with more than 3.94 million new stroke cases, 28.76 million prevalent stroke patients and 2.19 million stroke-related deaths in 2019 in China [1]. For a stroke event, the length of stay is over 10 days and the cost is over $2000 [2]. Stroke creates a heavy burden on Chinese society and families [2]. Stroke due to basilar artery occlusion (BAO) is usually associated with poor prognosis [3]. BAO refers to the blockage of blood supply in the basilar artery due to in situ thrombosis caused by atherosclerosis or emboli migrating from other organs, frequently accompanied by an acute ischemic stroke. The basilar artery is one of the most vital intracranial arteries, supplying oxygenated blood to critical structures such as the brainstem, cranial nerves, and cerebellum. The best medical management (BMM) for BAO typically involves the administration of intravenous thrombolysis (IVT), antiplatelet medications, anticoagulants, or a combination of these treatments, in accordance with the 2019 American Heart Association guidelines for stroke management [4]. For strokes caused by BAO presenting with moderate-to-severe clinical demonstration, over 80% of patients die or survive with a severe disability even though the BMM is given [5–7].

Mechanical thrombectomy (MT) using stent retrievers (SR) is now primary approach for managing acute stroke resulting from large-vessel occlusion in the anterior circulation [8]. Economic evaluations have revealed that MT is cost-effective for the treatment of acute stroke caused by large-vessel occlusion of the anterior circulation in many countries [9–12]. Owing to the benefits of MT in stroke patients with anterior circulation occlusion and its cost-effectiveness in such patients, MT is currently widely used in the treatment of anterior circulation occlusion in China [12,13]. Recently, ATTENTION showed that endovascular thrombectomy led to better functional outcomes at 90 days than the BMM for patients with BAO within 12 h after the estimated time of BAO [14]. Another head-to-head study, the BAOCHE, showed that for BAO stroke between 6 and 24 h after symptom onset, MT using SR was associated with better functional status at 90 days than BMM [15]. Notably, the above two studies were performed in Chinese patients with BAO, and patients in the intervention group of the BAOCHE study received MT using stent retrievers with a wider time window of 24 h.

Currently, the cost of SR is significantly higher than that of BMM [12]. A study conducted in China revealed that the adoption of MT using SR for Chinese patients led to an additional cost of $9821 in 2013. This cost encompassed the costs associated with the MT procedure, MT devices, and other related costs [12]. In contrast, the average cost of hospitalization for stroke patients without MT in China during 2013 amounted to approximately $2217 per occurrence [12]. However, the cost-effectiveness of SR in Chinese patients with BAO is yet to be investigated. Thus, this study aimed to evaluate the cost-effectiveness of SR, specifically in the context of Chinese patients with BAO.

## Methods

This study adhered to the reporting guidelines outlined in the 2022 Statement of the Consolidated Health Economic Evaluation Reporting Standards [16].

### Participants

This study focused on Chinese patients with BAO who experienced stroke symptoms within a time window of 6–24 h after symptom onset. In total, 217 patients were included in the BAOCHE study [15]. The mean patient age was 64 years. Approximately 47% of BAO sites were located in the proximal basilar artery, 36% in the middle basilar artery, and 17% in the distal basilar artery. Within Table 1, a comprehensive overview of the study cohort’s baseline characteristics is presented.

This was a modeling study, and ethical approval from the institutional review board was not applicable for this type of study.

**Table 1:** Baseline characteristics of the individuals enrolled in the BAOCHE trial.

Characteristic	Value	Reference
Patients number—no.	217	[15]
Mean age—year	64	[15]
Male—no. (%)	147 (74)	[15]
mRS score of 0 before stroke—no. (%)	174 (80)	[15]
NIHSS score distribution—no. (%)		
6–20	127 (59)	[15]
>20	90 (41)	[15]
Median SBP at hospital arrival—mmHg	155	[15]
Median glucose level at hospital arrival- mmol/L	7.8	[15]
Intravenous thrombolysis—no. (%)	38 (18)	[15]
Occlusion site—no. (%)		[15]
Proximal basilar artery	98 (47)	[15]
Middle basilar artery	77 (36)	[15]
Distal basilar artery	36 (17)	[15]
Median duration—min		
From stroke onset to randomization	663	[15]
From stroke onset to revascularization	790	[15]
From hospital admission to groin puncture	153	[15]
From groin puncture to revascularization	85	[15]

Abbreviations: BAOCHE. Basilar Artery Occlusion Chinese Endovascular Trial; mRS. modified Rankin Scale; NIHSS. National Institutes of Health Stroke Scale; SBP. Systolic Blood Pressure.

### Settings and comparators

The comparators in this study were SR plus BMM and BMM alone. All patients included in the study were admitted to acute stroke units or neurological intensive care units (NICU) and received treatment in accordance with the current Chinese guidelines for acute ischemic stroke management [17], which was similar to the 2019 AHA/ASA guideline for acute stroke [4]. Approximately 14% of the patients in the SR group received IVT, and this value was 21% in the BMM group.

### Model overview

We devised an integrated decision tree and Markov model to assess the cost-effectiveness of SR alongside BMM in comparison to BMM alone, from the viewpoint of the Chinese healthcare system. Within the decision tree framework, patients meeting the eligibility criteria for BAO were randomly assigned to either SR plus BMM or BMM alone. The observation period extended to 90 days (equivalent to 0.25 years). Following this, patients were transitioned into the Markov model, commencing from the state defined in the decision tree and progressing through subsequent cycles until reaching the endpoint of mortality. The simulation period encompassed 20 years, with each cycle lasting 3 months. In the BAOCHE study, the average age of the participants was 64 years, and the Markov model comprised 79 cycles (corresponding to 19.75 years). Notably, the simulation period was extended beyond the life expectancy of 78 years reported for China in 2021 [18]. The rationale behind selecting a lifetime horizon rather than relying solely on life expectancy stemmed from that life expectancy represents the average lifespan, although some patients may surpass this average. Hence, incorporating a time horizon that exceeds life expectancy provides a more comprehensive reflection of actual circumstances. It is important to emphasize that although some simulated patients may not survive until reaching their life expectancy, their termination is appropriately accounted for within the Markov model. All patients, irrespective of surpassing the life expectancy or not, were included in our analysis.

There were three transition states of “non-disabling stroke,” “disabling stroke,” “Recurrent stroke,” and an absorbed state of “Dead.” Patients had the potential to transition from one state to another or remain in the same state throughout the course of the study, but the process would conclude should the patient transition into the absorbed state.

**Fig 1:**
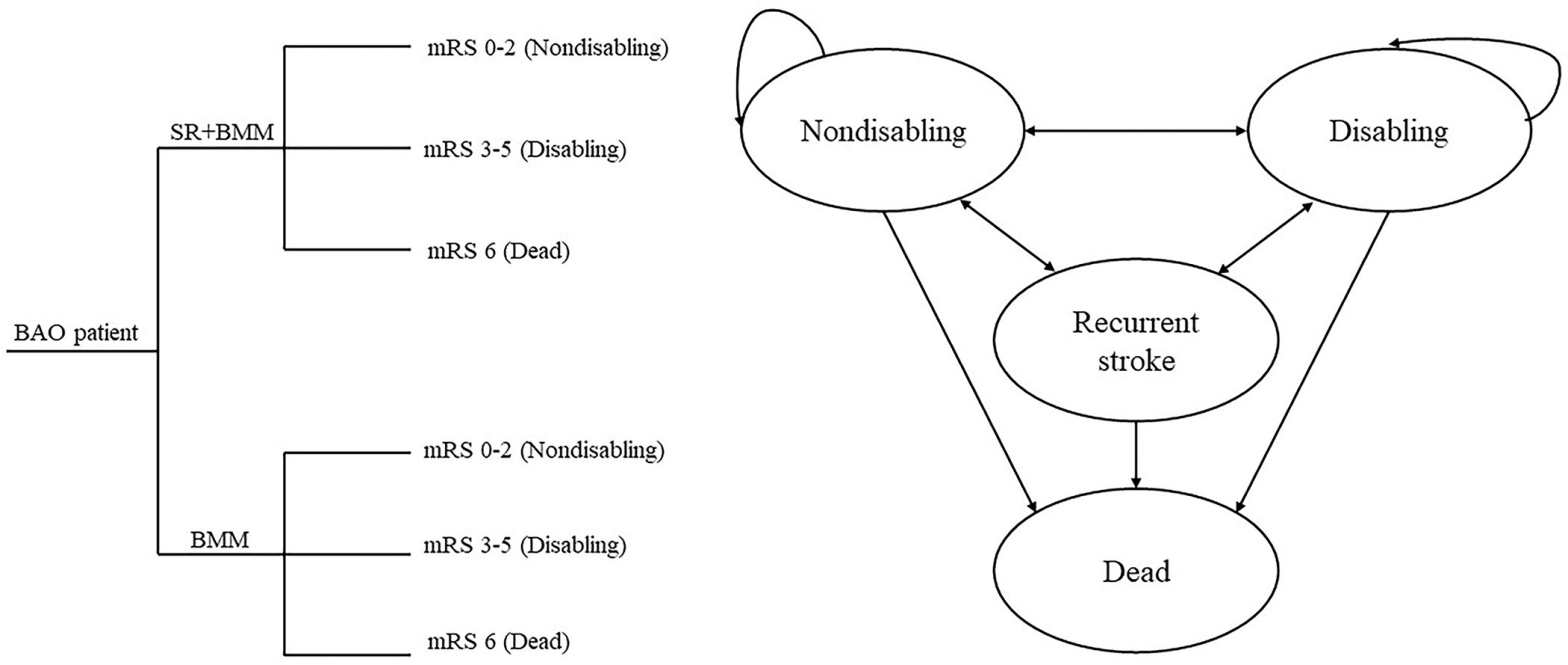
The schematic representation of the decision tree and Markov model. BAO, basilar artery occlusion; SR, stent retriever; BMM, best medical management; mRS, modified Rankin Scale.

The current investigation was undertaken from the viewpoint of the healthcare system in China, with particular emphasis on assessing direct medical costs. It is important to note that our analysis did not include direct nonmedical or indirect costs in the evaluation. The expenses within the decision tree and Markov model were combined to compute the overall cost, while the total effectiveness was determined using an analogous approach. The conversion of all costs to USD was based on the exchange rate of 6.4515 Chinese Yuan (CNY) to 1 USD [19]. Following the principles set forth by the China Guidelines for Pharmacoeconomic Evaluations, expenses occurring prior to 2021 and future costs were discounted at a rate of 0.05, with a variation between 0 and 0.08 [20]. Fig 1 displays a diagram representing the decision tree and Markov model, which has been substantiated in several previous studies [9,11,21,22].

### Model assumptions

Several assumptions were made to simulate cost and effectiveness in Chinese patients with BAO. First, the transition probability of clinical events for stroke caused by BAO in patients with the same mRS score was assumed to be equivalent to that for stroke caused by anterior circulation occlusion. This assumption is based on a cohort study that investigated long-term mortality after BAO. This study demonstrated that the long-term mortality rate in patients with BAO who survived the first 3 months corresponded to the large-artery stroke subtype of the anterior circulation. Secondly, it was assumed that “recurrent stroke” originating from "non-disabling stroke" has the potential to transition to "non-disabling stroke," "disabling stroke," or "dead." Conversely, recurrent strokes originating from "disabling stroke" can only transition to "disabling stroke" or "dead" and cannot lead to a more favorable outcome of "non-disabling stroke.” This assumption was based on the advice of neurologists. Third, the long-term outcomes were assumed to depend solely on the mRS scores at 3 months, regardless of whether SR was performed. Previous studies have adopted such assumptions [9,23,24].

### Input parameters

#### Clinical data

The effectiveness of SR plus BMM versus BMM alone in Chinese patients with BAO was obtained from the BAOCHE study. In the BAOCHE study, the proportion of “non-disabling stroke (mRS 0–2),” “disabling stroke (mRS 3–5),” and “dead (mRS 6)” at 90 days was 0.391, 0.300, and 0.309, respectively, in the SR plus BMM group, and the corresponding proportion was 0.140, 0.439, 0.421, respectively, in the BMM group (Table 2).

**Table 2:** Input parameters of clinical data.

Parameters	Value	Range	Source
Distribution of Functional Scores at 90 Days in SR			
mRS 0–2 (Non-disabling)	0.39091	0.29972–0.4821	[15]
mRS 3–5 (Disabling)	0.3	0.261–0.351	[15]
mRS 6 (Dead)	0.30909	0.22273–0.39545	[15]
Distribution of Functional Scores at 90 Days in BMM			
mRS 0–2 (Non-disabling)	0.14019	0.07440–0.20597	[15]
mRS 3–5 (Disabling)	0.43925	0.34521–0.53329	[15]
mRS 6 (Dead)	0.42056	0.32702–0.5141	[15]
RR for mRS 0–2	2.79	1.65–4.71	[8]
RR for mRS 3–5	0.68	0.48–0.98	[8]
RR for mRS 6	0.73	0.51–1.05	[8]
IVT proportion in SR	0.13636	0.07223–0.2005	[15]
IVT proportion in BMM	0.21495	0.13712–0.29279	[15]
Incidence of sICH in SR	0.05882	0.01316–0.10449	[15]
Incidence of sICH in BMM	0.01136	0–0.03351	[15]
Transition probabilities from month 3 to year 1			
Non-disabling to			
Non-disabling	0.955	/	[11]
Disabling	0.024	/	[11]
Recurrent stroke	0.013	/	[11]
Dead	0.008	/	[11]
Disabling			
Disabling	0.919	/	[11]
Non-disabling	0.029	/	[11]
Recurrent stroke	0.013	/	[11]
Dead	0.039	/	[11]
Transition probabilities after year 1			
Non-disabling			
Non-disabling	0.979	/	[11]
Disabling	0	/	[11]
Recurrent stroke	0.013	/	[11]
Dead	0.008	/	[11]
Disabling			
Disabling	0.948	/	[11]
Non-disabling	0	/	[11]
Recurrent stroke	0.013	/	[11]
Dead	0.039	/	[11]
Recurrent stroke come from a non-disabling stroke			
Non-disabling	0.834	/	[11]
Disabling	0.137	/	[11]
Dead	0.029	/	[11]
Recurrent stroke come from a disabling stroke			
Disabling	0.971	/	[25]
Dead	0.029	/	[25]
Annual background mortality in Chinese population			
60–64 years old	0.00734	/	[18]
65–69 years old	0.01218	/	[18]
70–74 years old	0.02081	/	[18]
75–79 years old	0.03604	/	[18]
80–84 years old	0.06503	/	[18]
85- years old	0.14828	/	[18]
RR of non-stroke death for mRS 3–5	1.68	1.49–1.92	[26]

Abbreviations: SR. stent retriever; mRS. modified Rankin Scale; BMM. Best medical management; IVT. Intravenous thrombolysis; sICH. symptomatic intracranial hemorrhage.

Due to the lack of accessible transition probability of patients diagnosed with BAO, we utilized transition probability data for stroke caused by anterior circulation occlusion in our Markov model. Although these probabilities were not directly derived from patients with BAO, they were suitable approximations for our analysis (10). In our Markov model, the 3-month transition probability remained consistent throughout the first three cycles and continued to be uniform for cycles beyond 1 year. However, it is important to note that the transition probabilities differed between the first three cycles and subsequent cycles, highlighting the distinction between these two periods. The transition probability was converted from the incidence rate with the formula of “3-month transition probability” = 1- exp (- “3-month incidence rate”) (Table 2).

#### Cost

In the BAOCHE study, the Solitaire device, a self-expanding stent that is retrievable and detachable, was employed for SR. The total cost of SR using the Solitaire device was $10,608 in 2019, and it would be inflated to $11,695 in 2021, considering the discount rate [13,27]. The cost of IVT was calculated using the same method, and the proportion of IVT was 14% and 21% in the SR and BMM groups, respectively (Table 2).

The cost of BMM for stroke treatment was extracted from the China National Stroke Registry [12,13], which included 21,902 consecutive Chinese stroke patients. This figure serves as an overall representation of the average cost of stroke treatment in China. However, it should be noted that these costs can vary based on the specific type of stroke. Specifically, for non-disabling stroke, the cost of BMM was $1,933; for disabling stroke, the cost of BMM was $2,640; and for stroke resulting in death, the cost of BMM was $2,138. For long-term annual healthcare costs, it was $1420 and $2182 for non-disabling stroke and disabling stroke, respectively. Annual costs were converted into quarterly costs to fit the model (Table 3).

**Table 3:** Input parameters of cost and utility.

Parameters	Value	Range	Source
Additional cost of SR ($)	11,695	10,059–13,520	[13,27]
Additional cost of IVT ($)	1720	848–2529	[28]
Annual cost for non-disabling stroke ($)	1420	1376–1465	[12,13]
Annual cost for Disabling stroke ($)	2182	1965–2323	[12,13]
Cost of BMM for non-disabling stroke ($)	1933	1905–1962	[12,13]
Cost of BMM for disabling stroke ($)	2640	2582–2699	[12,13]
Cost of BMM for stroke death ($)	2138	1965–2323	[12,13]
Cost of recurrent stroke ($)	1599	800–2399	[18]
Cost of sICH ($)	3507	3323–3700	[28]
Discount rate	0.05	0–0.08	[20]
Utility of non-disabling stroke	0.76	0.69–0.82	[12,28]
Utility of disabling stroke	0.21	0.17–0.26	[12,28]
Utility of recurrent stroke	0.42	0.11–0.71	[12,28]
Utility of dead	0	0	[12,28]

Abbreviations: SR. stent retriever; IVT. Intravenous thrombolysis; BMM. Best medical management; sICH. symptomatic intracranial hemorrhage.

#### Utility

The most significant factor influencing quality of life, the primary indicator of utility, was whether the stroke was disabling or non-disabling. Utility values for different stroke types were derived from a randomized controlled trial (RCT) conducted in China, yielding 0.76 for non-disabling stroke, 0.21 for disabling stroke, and 0.42 for recurrent stroke [12,29] (Table 3). Quality of life assessments were performed using the EuroQoL questionnaire, with the application of Chinese preference weights developed by Liu et al. [29].

There was a controversy surrounding the decision to discount utility [20]; in our primary analysis, the utility was subject to discounting, whereas in the scenario analysis, discounting of the utility was omitted.

#### Outcomes assessment

The outcome was the incremental cost-effectiveness ratio (ICER), which measured the additional cost per quality-adjusted life year (QALY). The willingness-to-pay (WTP) threshold for high cost-effectiveness was set at one per capita GDP, while the threshold for cost-effectiveness was set at three times per capita GDP[20]. If the ICER exceeded three times the per capita GDP, SR was deemed not cost-effective. If the ICER fell between one and three times the per capita GDP, it was considered cost-effective. ICER values below the per capita GDP threshold suggest a high level of cost-effectiveness for SR.

#### Scenario analysis

In previous studies, both the cost of MT and health utility had the most significant impact on the ICER [12,13,26], implying that fluctuations in these variables could render SR not cost-effective. Furthermore, the China Guidelines for Pharmacoeconomic Evaluations do not provide specific recommendations regarding the discounting of utility [20]. While our primary analysis relied on data from the BAOCHE trial [15] where SR was the exclusive method of MT, it is important to note that SR was also employed in patients with BAO in other trials assessing SR versus BMM [14,30,31], albeit not as the sole method. To account for these variations, we conducted a scenario analysis, considering factors such as not discounting utility, a 50% cost reduction or a 50% cost increase compared to current costs, and effectiveness based on the results of a meta-analysis.

### Sensitivity analysis

To assess the robustness of the findings, we conducted sensitivity analysis to account for result uncertainty. In the one-way sensitivity analysis, variations in input parameters caused fluctuations in the ICER, while we set the WTP threshold to evaluate result certainty. In the probabilistic sensitivity analysis (PSA), Monte Carlo simulation randomly sampled input parameters to model healthcare interventions, generating numerous simulations and forming a distribution of cost-effectiveness ratios [20]. Input parameters, such as cost, transition probability, and utility, followed specific probability distributions [20]. We performed 10,000 Monte Carlo iterations using PSA sampling to assess uncertainty. PSA outcomes were visually represented using a cost-effectiveness plane and an acceptability curve.

### Adverse events

Within the BAOCHE study, the occurrences of symptomatic intracranial hemorrhage (sICH) were recorded at 6% in the SR group and 1% in the BMM group [15]. The treatment cost for sICH was $3507 per case [12,13].

## Results

### Base case analysis

For Chinese patients with BAO, the total cost per patient over a 20-year period was $8678 if they received BMM alone. However, if they received SR plus BMM, the cost would increase to $21,988. The corresponding effectiveness outcomes were 1.45 QALY for BMM and 2.77 QALY for SR plus BMM. The ICER for SR plus BMM compared to BMM alone amounted to $10,050 per QALY, falling below the per capita GDP threshold of $12,551. If life quality was not taken into consideration, the corresponding effectiveness was 3.27 LY and 4.69 LY; thus, an ICER of $9385/LY was obtained (Table 4).

**Table 4:** Cost-effectiveness analysis results in the base-case and alternative scenario analysis.

Strategy	Total cost ($)	Incr-costs ($)	Total-eff (QALY)	Incr-eff (QALY)	ICER ($/QALY)	Total-eff (LY)	Incr-eff (LY)	ICER ($/LY)
***Base case***								
BMM	8678	/	1.45	/	/	3.27	/	/
SR+BMM	21,988	13,310	2.77	1.32	10,050	4.69	1.42	9385
***Scenario 1*. *Cost of SR reduced at 50%***								
BMM	8678	/	1.45	/	/	3.27	/	/
SR+BMM	16,141	7463	2.77	1.32	5635	4.69	1.42	5262
***Scenario 2*. *Cost of SR increased by 50%***								
BMM	8678	/	1.45	/	/	3.27	/	/
SR+BMM	27,836	19,158	2.77	1.32	14,466	4.69	1.42	13,508
***Scenario 3*. *Utility not discounted***								
BMM	8678	/	1.96	/	/	4.23	/	/
SR+BMM	21,988	13,310	3.83	1.88	7090	6.34	2.11	6309
***Scenario 4*. *Efficacy from meta-analysis***								
BMM	8488		1.71			3.35		
SR+BMM	21,560	13,072	2.65	0.94	13,903	4.45	1.10	11,902
Subgroup analysis								
Male								
BMM	8484	/	1.40	/	/	3.17	/	/
SR+BMM	21,731	13,247	2.68	1.28	10,346	4.54	1.37	9677
Female								
BMM	8874	/	1.49	/	/	3.38	/	/
SR+BMM	22,245	13,371	2.86	1.37	9777	4.84	1.47	9117
Age = 60 years old								
BMM	8913	/	1.51	/	/	3.40	/	/
SR+BMM	22,309	13,397	2.89	1.38	9699	4.88	1.48	9027
Age = 70 years old								
BMM	8137	/	1.31	/	/	2.98	/	/
SR+BMM	21,254	13,117	2.51	1.19	10,983	4.25	1.27	10,332

Abbreviations: Abbreviations: ICER. Incremental cost-effectiveness ratio; QALY. Quality-adjusted life year; LY. Life year; BMM. Best medical management; SR. stent retriever.

### Scenario analysis

In the scenario analysis considering undiscounted utility, the ICER was lower than the per capita GDP. Furthermore, when the cost of SR increased to 150% of the current price, the ICER was calculated as $14,466/QALY. Although slightly higher than the per capita GDP, it remained lower than threefold the per capita GDP benchmark. In the other scenarios analyzed, the ICER remained consistently lower than the per capita GDP (Table 4).

### Sensitivity analysis

**Fig 2:**
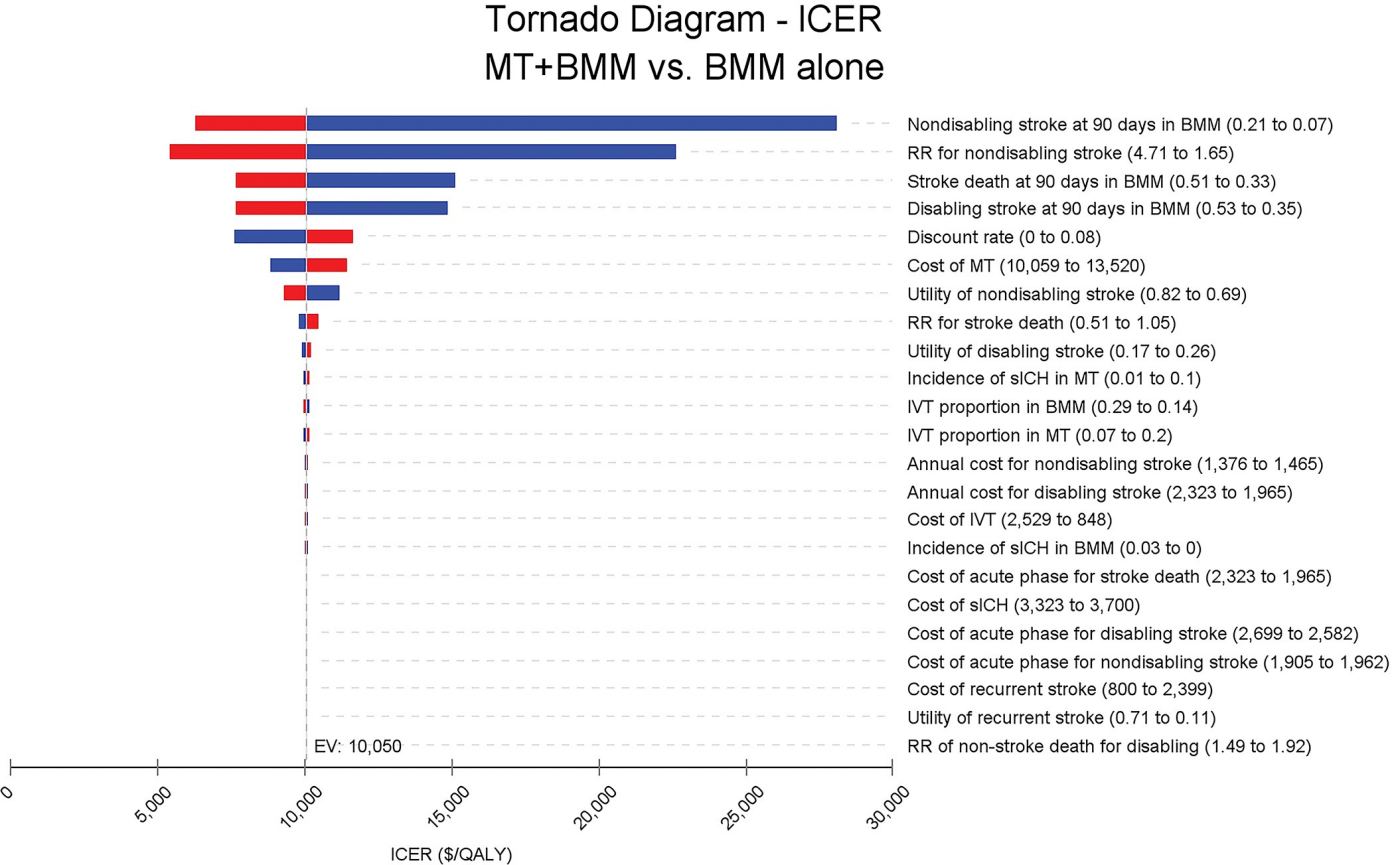
Tornado diagram of ICER of SR plus BMM versus BMM alone in Chinese patients with basilar-artery occlusion. Incidence of non-disabling stroke at day 90 impacted the largest on the ICER, and the risk ratio of non-disabling stroke impacted secondly to the ICER fluctuation. No parameters could lead to an ICER higher than $37,654/QALY. ICER, incremental cost-effectiveness ratio; SR, stent retriever, BMM, best medical management; sICH, symptomatic intracranial hemorrhage; IVT, intravenous thrombolysis; EV, expected value; WTP, willingness-to-pay; QALY, quality-adjusted life year.

Fig 2 demonstrates that the incidence of non-disabling stroke on day 90 had the most significant influence on the ICER, with the risk ratio of non-disabling stroke being the second most impactful factor on the ICER variation. The aforementioned two parameters could lead to an ICER higher than $12,551/QALY (the 2021 China’s GDP per capita) yet below $37,654/QALY (three times the per capita GDP).

**Fig 3:**
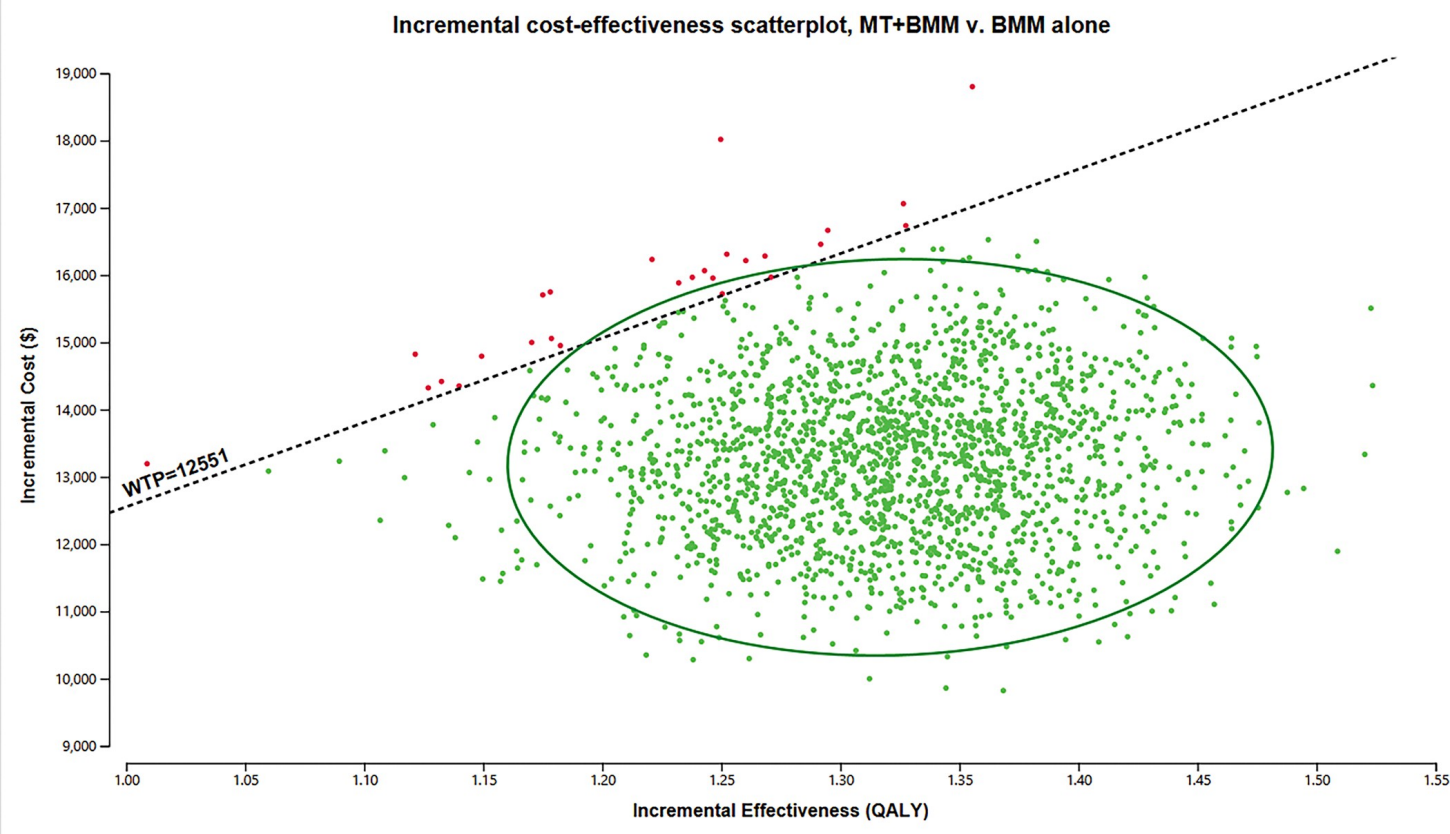
Cost-effectiveness plane. A WTP threshold of $12,551 per QALY was indicated by the dotted line. The cost-effectiveness plane displayed that over 98% of data points were situated below this WTP threshold line. SR: stent retriever; BMM: best medical management.

**Fig 4:**
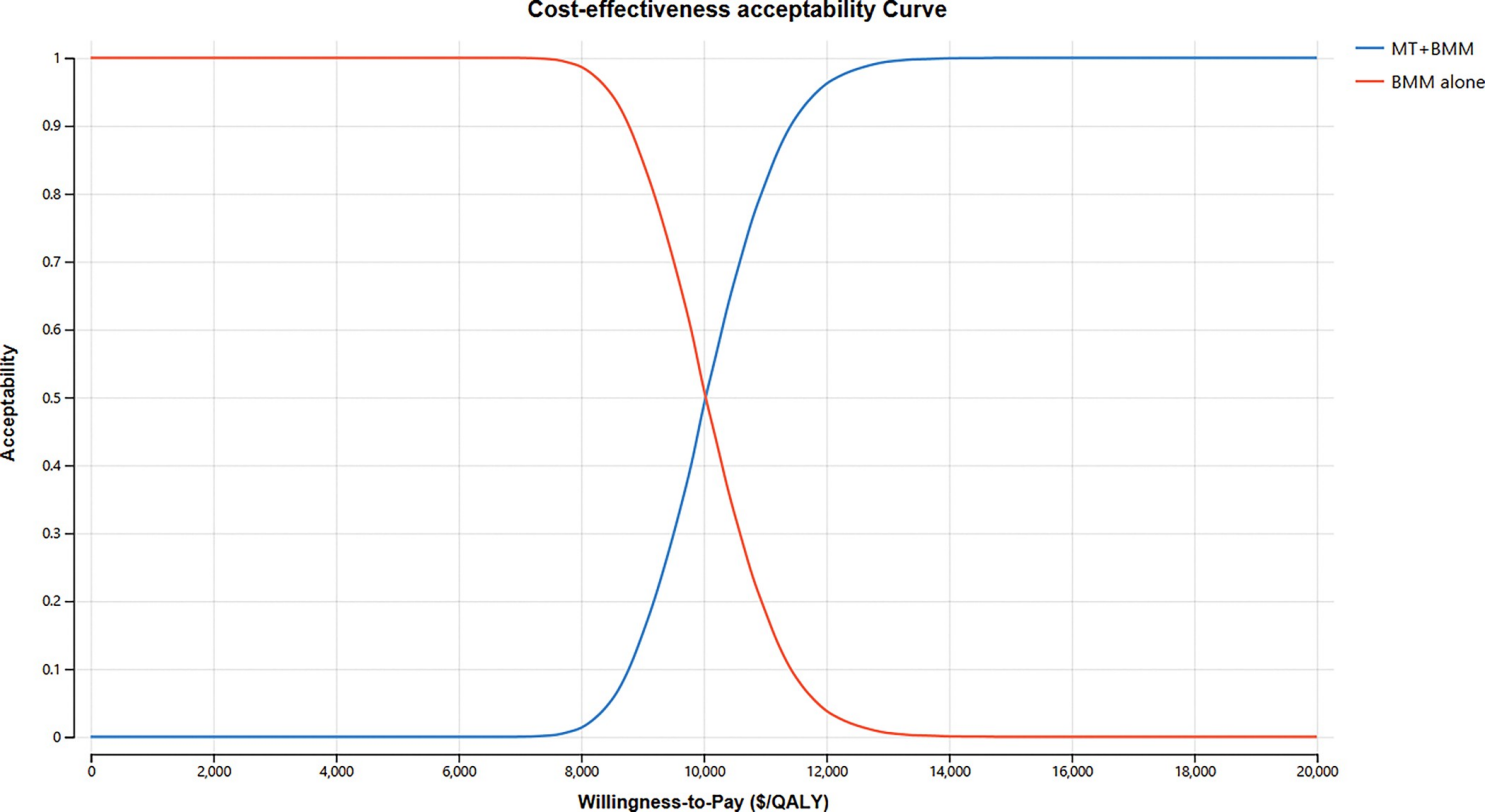
Cost-effectiveness acceptability curve. When the WTP threshold was $10,030/QALY, SR shared similar acceptability with BMM, and SR gained more acceptability than BMM when the WTP limit exceeded $10,030 per QALY. SR, mechanical thrombectomy; BMM, best medical management; WTP, willingness-to-pay; QALY, quality-adjusted life year.

**Fig 5:**
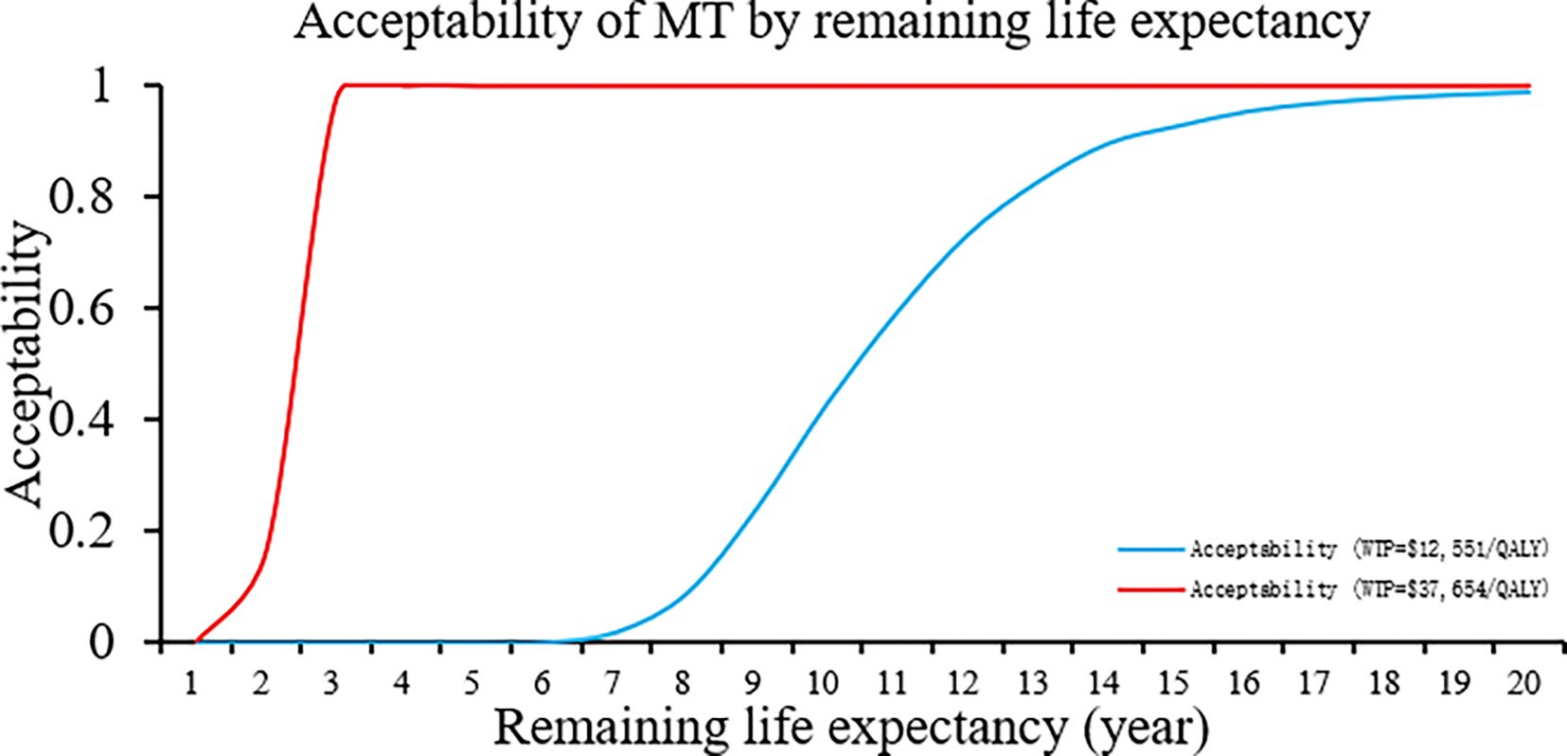
Acceptability curve of SR by remaining life expectancy. With a remaining life expectancy of no less than 3 years, SR was cost-effective for a patient with BAO. When the remaining life expectancy was over 10 years, SR was highly cost-effective.

PSA demonstrated that, at a WTP threshold of $12,551 per QALY, SR demonstrated remarkable cost-effectiveness, with a probability surpassing 98%. (Fig 3). The cost-effectiveness acceptability curve depicted that when the WTP threshold was established at $10,030 per QALY, SR exhibited a level of acceptability similar to that of BMM, and the acceptability was 0.9854 and 1 when the WTP threshold was set at one or three times the GDP in 2021 China (Fig 4). The acceptability curve based on the time horizon revealed that for Chinese patients with BAO with a remaining life expectancy of over 3 years, SR was considered cost-effective. Moreover, if the life expectancy exceeds 10 years, SR would be deemed highly cost-effective (Fig 5).

## Discussion

To the best of our knowledge, this is the first study to investigate the cost-effectiveness of SR in Chinese patients with BAO. Our findings indicate that SR is highly cost-effective in this patient population. From an economic standpoint, this study supports the use of SR in Chinese patients with BAO.

Currently, four RCTs have investigated the efficacy of MT or endovascular treatment (EVT) in patients with BAO [14,15,30,31]. The pioneering BEST study included Chinese patients with BAO and revealed no substantial evidence of variation in favorable outcomes between individuals treated with EVT and those receiving BMM alone, as assessed in the intention-to-treat analysis; however, the analysis of as-treated populations showed that EVT could improve the clinical prognosis [31]. The BASICS study, primarily carried out in Western nations, revealed no significant disparity between EVT and BMM in terms of favorable functional outcomes on day 90 [30,32]. Two other studies that mainly included Chinese patients with BAO found that MT or EVT was superior to BMM in these populations [14,15]. The time frame for EVT in the ATTENTION study was within 12 h after BAO, and it was 6–24 h in the BAOCHE study [14,15]. In addition to the above RCTs, the ATTENTION registry study indicated that EVT was associated with significantly better functional outcomes and survival at 90 days [7].

In our study, we employed data from the BAOCHE trial as our base-case analysis rather than conducting a meta-analysis of the aforementioned four RCTs [14,15,30,31], due to several key factors: the specific inclusion criteria in our study, focusing on patients experiencing stroke symptoms within a 6–24-hour time window, which only the BAOCHE trial satisfied; the exclusive use of SR as a procedural option in the BAOCHE trial, in contrast to the other three trials with aspiration as an alternative; and the utilization of cost data from the EAST study [27], featuring the Solitaire device, also the sole device in the BAOCHE trial, potentially offering a more accurate reflection of the treatment strategy’s cost.

Previous studies have consistently demonstrated the efficacy of using a SR in MT for anterior circulation occlusion [33,34]. Furthermore, the cost-effectiveness of this therapy has been confirmed in various countries and regions [10,11,23,26], even when performed >6 h after symptom onset [9,10]. In BAO cases, MT with a stent retriever is considered the primary therapeutic method. However, only one study investigated the cost-effectiveness of endovascular thrombectomy for acute BAO stroke from the perspective of the US healthcare system [35]. The study concluded that endovascular treatment was cost-effective for patients with BAO stroke, which aligns with our findings despite the differences in the Markov models used. Notably, the MT and stroke-related costs, including hospitalization and posthospitalization care, were significantly higher in their study than in ours. This finding adds to the robustness of our results. This study was the first to conduct a cost-effectiveness comparison between SR and the BMM approach in Chinese patients with BAO, providing insights from a healthcare system perspective. Our findings indicate that SR is highly cost-effective in the current Chinese healthcare settings.

BAO is associated with poor functional prognosis even when BMM is administered [3,36]. Although MT and EVT have significantly improved clinical prognosis, the mortality rate at 90 days was still higher than 30% [14,15,37]. Severe disability is common among survivors with poor recanalization; severe disability is common [31]. A study with a 20-year follow-up suggested a trend towards a higher rate of good and/or moderate outcomes during later years if patients were administered BMM [38]. These studies indicate that stroke-related deaths mainly occur in the acute phase rather than subsequent years after BAO. In patients with BAO, the 90-day mortality rates were 31% and 43% in the MT and BMM groups, respectively, indicating greater effectiveness in the MT group. In addition, non-disabling stroke was much higher in the MT group than in the BMM group, and the absolute risk reduction was 25%, leading to more effectiveness and less cost, as non-disabling stroke was associated with a lower cost of rehabilitation or drug use.

For several reasons, SR is highly cost-effective in Chinese patients. First, SR provides great benefits to patients with BAO. It could be observed that the effectiveness was 1.45 QALY in the BMM group but 2.77 in the SR group, which was mainly due to the reduction of mortality and disabling stroke in the SR group. Second, the cost of SR was lower in China than in other regions at $9427 in 2013 China and $13,803 in 2014 UK. Third, the mean age of Chinese patients with BAO was not very high, at 66 years in the ATTENTION study and 64 years in the BAOCHE study; the remaining life expectancy was over 10 years if a patient with BAO survived the stroke event. Currently, the average life expectancy in China exceeds 78 years, and a patient with BAO might benefit greatly from SR.

### Limitations

Based on the utilization of clinical data obtained exclusively from Chinese patients with BAO and cost data derived from domestic sources, our study results are robust. However, this study had some limitations. First, the conclusion of the present study was drawn from a mathematical model, and a real-world study might provide stronger clinical evidence [20]. Second, our study focused on a healthcare system perspective. However, adopting a societal viewpoint, which encompasses direct non-medical and indirect costs, may provide a more comprehensive assessment [20]. Several studies explored MT vs. BMM cost-effectiveness from multiple perspectives, revealing varying ICERs but consistent conclusions [39–41]. Third, the parameters used in our study were specific to Chinese patients or studies conducted within Chinese healthcare settings, which limits the generalizability of our conclusions to other countries. Our findings can be primarily applied to China and other regions with similar healthcare settings. Furthermore, our ability to perform subgroup analysis based on baseline characteristics was constrained by the unavailability of raw data from the BAOCHE study [15], which may have affected the robustness of our results. Additionally, although studies have indicated comparable recovery outcomes between BAO and anterior circulation cases with the same mRS scores [38], direct clinical data specifically from BAO cases would have been preferable.

## Conclusion

In conclusion, our study indicates that for Chinese patients with BAO, SR stands as a cost-effective intervention option, surpassing a 98% probability of cost-effectiveness at the current WTP threshold of $12,551 per QALY. Moreover, as MT technology advances, the cost of SR is likely to decrease, while the acceptability of this treatment may rise. These findings offer crucial insights for clinical decision-makers and healthcare policy developers. Nonetheless, it is imperative to acknowledge the need for real-world studies to validate our conclusions, ensuring their applicability and robustness in practical clinical settings.

## Supporting information

S1 Table Annual background mortality in Chinese population. (DOC)

S1 File Search strategy of meta-analysis. (DOC)

S1 Fig Flow chart of screening for inclusion. (DOC)

S2 Fig S2-S4 Figs. Forest plot of results of meta-analysis.
(DOC)
