# Supplementary material for: Economic evaluation of stent retrievers in basilar artery occlusion: An analysis from Chinese healthcare system perspective
Source: PLoS One. 2023 Nov 30;18(11):e0294929. doi: 10.1371/journal.pone.0294929 (PMC10688905; doi:10.1371/journal.pone.0294929)
Supplement: S1 Table — (DOC) [file pone.0294929.s001.doc]

**sTable1. Annual background mortality in Chinese population**

|  | **Mortality** | **Source** |
| --- | --- | --- |
| Male |  |  |
| 60-64 years old | 0.010193167 | China Health Statistics Yearbook |
| 65-69 years old | 0.016358043 | China Health Statistics Yearbook |
| 70-74 years old | 0.026896512 | China Health Statistics Yearbook |
| 75-79 years old | 0.045101058 | China Health Statistics Yearbook |
| 80-84 years old | 0.077240888 | China Health Statistics Yearbook |
| 85- years old | 0.163453387 | China Health Statistics Yearbook |
| Female |  |  |
| 60-64 years old | 0.004466809 | China Health Statistics Yearbook |
| 65-69 years old | 0.008175163 | China Health Statistics Yearbook |
| 70-74 years old | 0.015091107 | China Health Statistics Yearbook |
| 75-79 years old | 0.028177245 | China Health Statistics Yearbook |
| 80-84 years old | 0.055507109 | China Health Statistics Yearbook |
| 85- years old | 0.138390632 | China Health Statistics Yearbook |
