# Supplementary material for: Economic evaluation of stent retrievers in basilar artery occlusion: An analysis from Chinese healthcare system perspective
Source: PLoS One. 2023 Nov 30;18(11):e0294929. doi: 10.1371/journal.pone.0294929 (PMC10688905; doi:10.1371/journal.pone.0294929)
Supplement: S1 File — (DOC) [file pone.0294929.s002.doc]

**Search strategy of Cochrane**

ID Search Hits

#1 (endovascular treatment):ti,ab,kw (Word variations have been searched) 3563

#2 (mechanical thrombectomy):ti,ab,kw (Word variations have been searched) 795

#3 (vertebrobasilar artery occlusion):ti,ab,kw (Word variations have been searched) 41

#4 (basilar artery occlusion):ti,ab,kw (Word variations have been searched) 171

#5 #1 or #2 4097

#6 #3 or #4 198

#7 #5 and #6 110

**Search strategy of Pubmed**

(((endovascular treatment[Title/Abstract]) OR (mechanical thrombectomy[Title/Abstract])) AND ((vertebrobasilar artery occlusion[Title/Abstract]) or (basilar artery occlusion[Title/Abstract]))) and ("7502039"[Journal])

**Search strategy of Embase**

#7 AND #5 #6

#6 OR #3 #4

#5 OR #1 #2

#4 'basilar artery occlusion':ab,ti

#3 'vertebrobasilar artery occlusion':ab,ti

#2 'mechanical thrombectomy':ab,ti

#1 'endovascular treatment':ab,ti
