# Supplementary figures and images for: Economic evaluation of stent retrievers in basilar artery occlusion: An analysis from Chinese healthcare system perspective
Source: PLoS One. 2023 Nov 30;18(11):e0294929. doi: 10.1371/journal.pone.0294929 (PMC10688905; doi:10.1371/journal.pone.0294929)

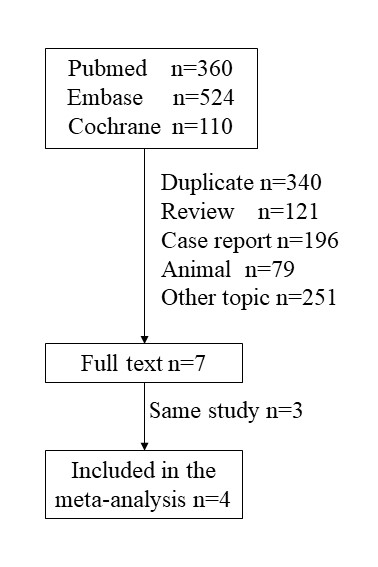


sFigure 1. Flow chart of screening for inclusion

Supplement: S1 Fig — (DOC) [file pone.0294929.s003.doc]
