# Supplementary material for: Economic evaluation of stent retrievers in basilar artery occlusion: An analysis from Chinese healthcare system perspective
Source: PLoS One. 2023 Nov 30;18(11):e0294929. doi: 10.1371/journal.pone.0294929 (PMC10688905; doi:10.1371/journal.pone.0294929)
Supplement: S2 Fig — Forest plot of results of meta-analysis. (DOC) [file pone.0294929.s004.doc]

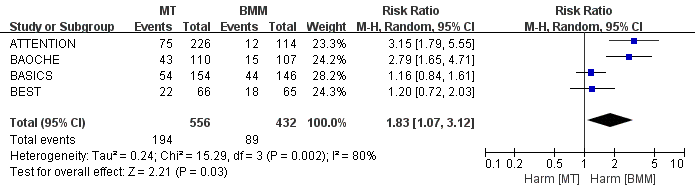


sFigure 2. Forest plot of excellent outcomes of MT versus BMM


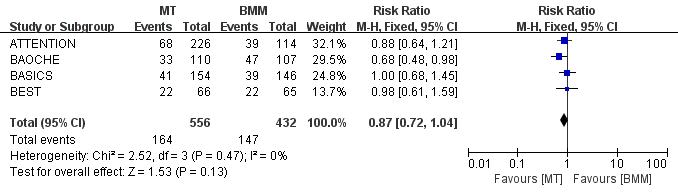


sFigure 3. Forest plot of mRS 3-5 of MT versus BMM


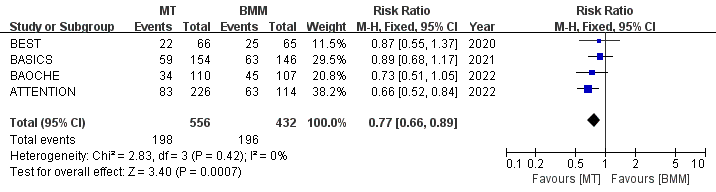


sFigure 4. Forest plot of death of MT versus BMM
